# Medical Emergency Preparedness in Primary Dental Care Following Practical Training: An Urban–Rural Comparison

**DOI:** 10.3390/healthcare14213695

**Published:** 2026-11-01

**Authors:** Mirko Tomić, Ivana Šutej, Augustin Pintarić, Josip Bošnjak, Matej Črep, Marko Mornar Jelavić, Hrvoje Pintarić

**Affiliations:** 1 Zagreb County Health Center, 10 430 Samobor, Croatia; 2 School of Dental Medicine, University of Zagreb, 10 000 Zagreb, Croatia; isutej@sfzg.hr (I.Š.);; 3 School of Medicine, University of Zagreb, 10 000 Zagreb, Croatia; 4 Polyclinic Medikol, 10 000 Zagreb, Croatia; mjelavic@yahoo.com; 5 Sestre Milosrdnice University Hospital Center, 10 000 Zagreb, Croatia

**Keywords:** dentists, dental practice, medical emergencies, emergency preparedness, continuing education, rural health, urban health

## Abstract

**Background/Objectives:** Medical emergencies in dental practice require adequate professional preparedness and access to appropriate resources. This study assessed changes in dentists’ self-perceived preparedness and reported availability of emergency medications and equipment one month after practical training, with an exploratory comparison between urban and rural institutions. **Methods:** This prospective, single-group pre–post study included 42 dentists from two Croatian primary healthcare institutions (18 urban and 24 rural). No control group was included. Participants completed the same questionnaire before and one month after a standardized medical emergency workshop. Paired changes were analysed using the Wilcoxon signed-rank test, and between-group differences using the Mann–Whitney U test. Changes in individual resources were assessed using exact McNemar tests with Holm adjustment. **Results:** Self-perceived educational adequacy increased from a median of 2 [IQR 2–3] to 4 [IQR 3–4] (*p* < 0.001), with improvement reported by 33 participants (78.6%). The mean paired increase in self-perceived educational adequacy was 1.19 points (95% CI: 0.93–1.48). In an exploratory comparison, the increase was greater in the rural than in the urban institution (between-group difference in mean change: 1.11 points; 95% CI: 0.68–1.53; *p* < 0.001). Reported medication availability increased from a median of 3 [IQR 2–4] to 4 [IQR 3–4.75] (*p* < 0.001), whereas perceived adequacy of medication supply did not change significantly (*p* = 0.415). Neither perceived equipment adequacy nor the number of reported equipment items changed significantly. **Conclusions:** One month after training, dentists reported improved self-perceived educational adequacy and increased reported availability of emergency medications, without corresponding significant improvements in perceived medication adequacy or equipment preparedness. These findings highlight the distinction between perceived preparedness and reported resources, although the absence of a control group precludes causal conclusions.

## 1. Introduction

Medical emergencies may occur unexpectedly during dental treatment and, although severe events are relatively uncommon [1], they can have serious consequences if they are not recognized and managed promptly [2,3]. Syncope remains the most frequently reported medical emergency in dental practice, while hypoglycemia, anxiety-related events, adverse reactions to local anesthetics, seizures, cardiovascular events and other acute conditions are also encountered in routine care [4,5,6,7]. The increasing number of older patients and patients with multiple chronic diseases further emphasizes the need for dental professionals to maintain adequate preparedness for medical emergencies [8].

Preparedness for medical emergencies in dental practice depends not only on theoretical knowledge, but also on the ability to recognize clinical deterioration, initiate appropriate emergency procedures and use the necessary medications and equipment [2,8,9,10,11]. Previous studies have repeatedly shown that a substantial proportion of dentists do not feel sufficiently confident in managing medical emergencies or performing cardiopulmonary resuscitation, despite having encountered such events in clinical practice [2,3,11,12,13,14,15,16]. Inadequate availability of emergency equipment and medications has also been reported, suggesting that preparedness should be considered as a combination of professional competence and practice-level resources rather than as an individual clinical skill alone [2,11,13,16].

Practical and simulation-based training has therefore become an important component of continuing education in medical emergency management. Hands-on training can improve knowledge, confidence and practical skills, whereas deterioration of previously acquired skills over time supports the need for regular refresher training [8,17,18,19,20,21,22]. However, educational interventions may influence different aspects of preparedness at different rates [23,24]. Perceived educational adequacy and awareness may change shortly after training, whereas changes in practice-level resources may be constrained by organisational and financial barriers [24,25].

The organisation and resources of dental practices may also vary according to the healthcare setting in which they operate. Although urban–rural differences in access to and utilisation of dental care have been described in different healthcare systems [26,27], evidence directly comparing preparedness for medical emergencies between urban and rural dental practices remains limited and context-dependent [28,29]. Such differences may be particularly relevant in primary dental care, where rural settings may experience longer access and transport times to external emergency medical services [30]. In Croatia, regulations define minimum requirements for dental practices, including provisions for the management of allergic reactions and oxygen delivery, but do not provide a comprehensive list of medications and devices for the full spectrum of medical emergencies encountered in dental practice [31].

A recent Croatian national study by Špiljak et al. [29] identified considerable variability in dentists’ self-perceived readiness to manage medical emergencies and in the availability of emergency medications and equipment. The study also demonstrated differences associated with professional characteristics, previous training and healthcare settings. However, its cross-sectional design did not allow assessment of changes in preparedness following practical training. Although previous educational studies have evaluated changes in knowledge, confidence and practical performance, evidence regarding short-term changes in both self-perceived preparedness and reported practice-level resources remains limited, particularly in relation to different geographical settings.

The present study aimed to assess changes in dentists’ self-perceived adequacy of education and reported availability of emergency medications and equipment before and one month after a practical medical emergency workshop in two Croatian primary healthcare institutions. Changes in perceived adequacy of medication and equipment supply were also examined. The comparison between the urban and rural institutions was planned from the outset as part of the doctoral research protocol. The principal outcome of the present analysis was the change in self-perceived adequacy of education, while changes in perceived medication and equipment adequacy and reported resource availability were evaluated as additional outcomes. However, given the inclusion of only one institution per setting and the absence of prespecified directional hypotheses, these comparisons were considered exploratory rather than confirmatory.

## 2. Materials and Methods

### 2.1. Study Design, Participants and Ethics Committee

This prospective, uncontrolled before-and-after study was conducted between September 2023 and September 2024 among dentists working in two Croatian primary healthcare institutions: Health Centre Zagreb–Centar, representing the urban setting, and Health Centre Krapina-Zagorje County, representing the rural setting.

The institutions were selected by convenience based on organisational feasibility and geographical accessibility. Health Centre Zagreb–Centar was selected as the largest of the three public health centres in Zagreb, while the institution in Krapina-Zagorje County was selected because of its proximity to Zagreb, facilitating the organisation of the educational workshop.

Eligible participants were dentists registered in the national Register of Dentists, actively practising in primary dental care and holding a valid licence for independent clinical practice. Retired dentists, those not directly involved in patient care and those without a valid licence were excluded. All eligible dentists working within the two participating institutions were invited to participate voluntarily. A total of 42 dentists were enrolled, including 18 from the urban and 24 from the rural institution. All participants completed both the baseline and one-month follow-up assessments. The individual dentist was the unit of analysis. Each participant reported on their own dental practice, with no dental practice represented by more than one participant.

The participating institutions were not selected to provide representative samples of urban and rural dental practices. As each geographical setting was represented by a single institution, differences between settings could not be separated from institution-specific characteristics. Accordingly, urban–rural comparisons were considered exploratory.

The broader domains of educational preparedness and emergency resource availability were defined in the original research protocol, whereas the five specific outcomes were operationalized for the present analysis.

The study was approved by the Ethics Committee of the School of Dental Medicine, University of Zagreb, Croatia, at its meeting held on 15 June 2023, with the formal approval decision issued on 16 June 2023 (approval No. 05-PA-30-19-6/2023). All participants provided written informed consent before participation.

### 2.2. Questionnaire and Educational Workshop

The 19-item questionnaire was developed specifically for this study by an interdisciplinary team comprising a professor of emergency medicine, a cardiologist, and a dentist specializing in pharmacology. Prior to data collection, the preliminary questionnaire was pilot-tested by five dentists affiliated with the School of Dental Medicine who were not included in the study sample. They were asked to assess the clarity and relevance of the questions and provide feedback. Based on their comments, two questions were revised and three additional questions were incorporated into the final version. The pilot testing was intended to improve the clarity and relevance of the questionnaire rather than establish its psychometric properties. No formal validity or reliability testing was performed.

Participants completed the questionnaire immediately before the workshop and again one month later. It collected demographic and professional information, previous experience with medical emergencies and prior emergency training, as well as reported availability of emergency medications and equipment. Participants also rated the adequacy of their education, emergency equipment and medication supply on a five-point scale, ranging from 1 (insufficient) to 5 (excellent). Higher scores indicated greater perceived adequacy. The complete questionnaire is provided as Supplementary File S1.

The questionnaire included eight equipment items: an automated external defibrillator, pulse oximeter, Magill forceps, self-inflating bag with mask, oropharyngeal airway, endotracheal tube, laryngoscope and oxygen cylinder. The nine medication items were adrenaline, bronchodilators, antihistamines, nitroglycerin, benzodiazepines, acetylsalicylic acid, glucose, corticosteroids and oxygen. The reported numbers of available equipment items (0–8) and medications (0–9) were calculated as the sum of affirmative responses within each category. Oxygen was included separately as an equipment item (oxygen cylinder) and a medication item, reflecting the questionnaire structure.

The educational intervention consisted of two workshops, one at each participating institution, attended by 18 urban and 24 rural dentists, respectively. The theoretical component lasted approximately 45–60 min and covered the recognition and initial management of medical emergencies in dental practice, including pharmacological management. This was followed by 1–2 h of supervised practical training in basic life support, cardiopulmonary resuscitation, airway management, management of choking and intravenous access, using manikins and appropriate training equipment.

Both workshops were coordinated by a professor of cardiology certified in emergency care. The pharmacological component was delivered by a professor of pharmacology, while practical training was supported by medical doctors affiliated with the StEPP association. The same educational team delivered both workshops using identical curricula, presentations and training equipment. Participants attended each workshop as a single group, without division into smaller subgroups, and individually practised emergency procedures under supervision.

Participants independently completed the baseline questionnaire before the workshop, without assistance from the instructors. Follow-up responses were collected one month later and matched to baseline responses using unique participant identification codes. The presentations were made available by email upon request. Participants were not instructed or required to review, purchase or supplement emergency medications or equipment during the follow-up period.

### 2.3. Statistical Analysis

Categorical variables were presented as frequencies and percentages, while continuous and ordinal variables were summarized using medians and interquartile ranges (IQRs). Baseline differences between the urban and rural groups were examined using the Mann–Whitney U test for continuous and ordinal variables and Fisher’s exact test or the chi-square test, as appropriate, for categorical variables.

Changes between baseline and one-month follow-up were assessed using the Wilcoxon signed-rank test for self-perceived adequacy of education, equipment and medication supply, as well as for the reported numbers of available emergency medications (0–9) and equipment items (0–8). The numbers of participants showing improvement, no change or deterioration were also reported for each outcome. Zero-change observations were retained when describing the numbers of participants with improved, unchanged, or worsened scores. For rank-based tests, tied observations and zero differences were handled according to the specified statistical procedures implemented in the analysis software.

Individual change scores were calculated as follow-up minus baseline values. Exploratory comparisons of change scores between the urban and rural groups were performed using the Mann–Whitney U test. Mean paired changes and between-group differences in mean change were additionally expressed with bootstrap 95% confidence intervals to describe the magnitude and precision of observed changes.

Changes in the reported availability of individual emergency medications and equipment items were evaluated using exact McNemar tests. Holm’s adjustment for multiple comparisons was applied separately within the nine medication items and eight equipment items. The questionnaire included three domains of self-perceived adequacy (education, medication supply, and equipment) and two categories of reported resource availability (medications and equipment). Item-level comparisons and adjustments for multiple testing were conducted as exploratory analyses during manuscript revision and were not prespecified as confirmatory analyses. Discordant pairs were reported to distinguish newly reported from no longer reported resources.

All tests were two-sided, with *p* < 0.05 considered statistically significant. No formal a priori sample size calculation was performed. The sample size was determined pragmatically by the number of eligible dentists who voluntarily participated in the educational workshops at the two selected institutions. This approach allowed paired assessment of changes within the participating cohort but was not intended to provide a statistically powered or representative comparison between urban and rural settings. Complete paired data were available for all 42 participants across the five main outcomes, with no missing item-level responses for the analyzed medication and equipment categories. The sample comprised dentists who voluntarily participated in the workshops and completed both assessments. The exact number of eligible dentists invited to participate could not be reliably established; therefore, a formal participation rate was not calculated. Given the limited sample size, the absence of a control group and the inclusion of only one institution per geographical setting, all urban–rural comparisons were considered exploratory. Statistical analyses were performed using MedCalc Statistical Software version 12.7.0.0 for Windows (MedCalc Software Ltd., Ostend, Belgium).

## 3. Results

### 3.1. Participant Characteristics and Previous Experience with Medical Emergencies

A total of 42 dentists completed both the baseline and one-month follow-up assessments, including 18 from the urban and 24 from the rural institution. The groups were comparable in age and professional experience. Previous medical emergency education during dental school was reported by 38.1% of participants, while 21.4% reported having attended a medical emergency training course before the intervention (Table 1).

At baseline, 22 participants (52.4%) reported having encountered at least one medical emergency during their clinical practice. Syncope was the most frequently reported event, followed by anxiety-related events and reactions associated with local anaesthetics. The types of previously encountered medical emergencies are presented in Supplementary Table S1.

Status epilepticus, hypoglycaemia, hypertensive crisis and chest pain were reported less frequently. These variables were used to describe the participants’ previous clinical experience and were not considered outcomes of the educational intervention.

### 3.2. Changes in Self-Perceived Preparedness and Reported Resource Availability

Changes in self-perceived educational preparedness and reported practice-level resources one month after the workshop are presented in Table 2.

For self-perceived educational adequacy, 9 participants remained unchanged and none worsened. For medication supply adequacy, 25 remained unchanged and 8 worsened; for reported medication counts, 27 remained unchanged and none decreased. For equipment adequacy, 29 remained unchanged and 8 worsened; for reported equipment counts, 39 remained unchanged and none decreased.

The most pronounced change was observed in self-perceived educational adequacy, which increased from a median of 2 [IQR 2–3] at baseline to 4 [IQR 3–4] at follow-up (*p* < 0.001). Overall, 33 participants (78.6%) reported an improvement, while nine (21.4%) reported no change and none reported deterioration.

A different pattern was observed for medication preparedness. The number of emergency medications reported as available increased from a median of 3 [IQR 2–4] to 4 [IQR 3–4.75] (*p* < 0.001), with 15 participants (35.7%) reporting an increase and 27 (64.3%) reporting no change. In contrast, self-perceived adequacy of medication supply showed no statistically significant change, despite an increase in the median score from 2 [IQR 2–3] to 3 [IQR 2–3] (*p* = 0.415).

Emergency equipment showed little change during follow-up. The median number of reported available equipment items was 0 [IQR 0–0] at baseline and 0 [IQR 0–1] at follow-up (*p* = 0.083). Only three participants (7.1%) reported an increase in the number of available equipment items. Self-perceived adequacy of emergency equipment remained unchanged, with a median score of 1 [IQR 1–2] at both assessments (*p* = 0.499).

Changes in the reported availability of individual emergency medications and equipment items are presented in Supplementary Table S2. After adjustment for multiple comparisons, corticosteroids were the only individual medication showing a statistically significant increase in reported availability (Holm-adjusted *p* = 0.035). No individual equipment item showed a statistically significant change after adjustment.

### 3.3. Urban–Rural Differences in Changes over Time

Exploratory comparisons of changes between the urban and rural groups are presented in Table 3.

Self-perceived adequacy of education increased in both settings, with a greater change observed among dentists in the rural institution. The mean individual increase was 0.56 points in the urban group and 1.67 points in the rural group. The between-group difference in mean change was 1.11 points (95% CI: 0.68–1.53; Mann–Whitney U test, *p* < 0.001).

At baseline, dentists in the rural institution reported a higher number of available emergency medications and equipment items than those in the urban institution. The mean number of reported medications was 4.00 in the rural group and 2.67 in the urban group (*p* = 0.0046), while the corresponding mean numbers of equipment items were 0.75 and 0.11 (*p* = 0.0196).

Despite these baseline differences, changes in reported medication availability did not differ significantly between the two groups (*p* = 0.893). Similarly, no statistically significant urban–rural difference was observed in changes in equipment availability (*p* = 0.409).

No statistically significant between-group differences in changes were observed for self-perceived adequacy of emergency equipment (*p* = 0.544) or medication supply (*p* = 0.897).

These comparisons were considered exploratory because each geographical setting was represented by a single healthcare institution.

## 4. Discussion

This study evaluated changes in dentists’ self-perceived preparedness and reported availability of emergency medications and equipment one month after a practical medical emergency workshop in two Croatian primary dental care institutions. The most prominent finding was an increase in self-perceived educational adequacy, accompanied by an increase in the reported number of available emergency medications. In contrast, neither perceived adequacy of medication supply nor perceived adequacy and reported availability of emergency equipment changed significantly. Although the increase in perceived educational adequacy was greater among dentists working in the rural setting, changes in the availability of medications and equipment did not differ between the urban and rural groups. These findings indicate that different dimensions of emergency preparedness may show different patterns of change over a short follow-up period. However, given the uncontrolled before-and-after design, the observed changes cannot be attributed directly to the educational intervention.

The observed increase in self-perceived educational adequacy is consistent with studies reporting improvements in confidence and perceived preparedness following simulation-based emergency training among dental professionals [23,25]. Similar benefits of structured resuscitation training have also been reported in other healthcare and educational settings [17,18,19,20]. Opportunities to practise emergency procedures are particularly relevant in dental practice because severe medical emergencies are relatively uncommon, limiting routine clinical exposure [2,3]. Nevertheless, self-perceived educational adequacy should not be equated with objectively assessed knowledge or clinical competence. The present study did not include practical performance assessments or standardized knowledge tests, and therefore cannot establish whether the observed changes translated into improved emergency management skills. Furthermore, the one-month follow-up does not provide information on long-term retention of these skills, which has been identified as an important limitation of emergency training programmes [18,20,23].

An important finding was the discrepancy between reported medication availability and perceived adequacy of medication supply. Although the number of medications reported as available increased, participants did not report a statistically significant improvement in their overall assessment of medication preparedness. This distinction is important because the presence of individual medications does not necessarily indicate that an emergency kit is sufficiently equipped or that practitioners are confident in using its contents. Previous research has similarly demonstrated differences between reported resource availability and self-perceived preparedness [1,13]. One possible explanation for our finding is that the workshop increased participants’ awareness of the medications already present in their practices, rather than necessarily leading to additional procurement. Alternatively, some medications may have been obtained during the follow-up period. Since availability was self-reported and no physical verification or documentation of purchases was performed, these explanations cannot be distinguished.

At the individual medication level, corticosteroids showed the most consistent increase in reported availability and were the only medication remaining statistically significant after adjustment for multiple comparisons. This finding should be considered exploratory and should not be interpreted as evidence of improved clinical management of medical emergencies. Medication availability alone does not establish the appropriateness of the emergency kit, particularly because individual medications differ substantially in their clinical relevance, indications, and required administration skills. An effective response to medical emergencies requires not only appropriate resources but also knowledge, practical competence, and established management protocols [2,8]. Therefore, although the observed increases in self-perceived educational adequacy and reported medication availability were statistically significant, their practical relevance remains uncertain in the absence of objective assessments of clinical competence, medication appropriateness, and readiness to manage actual emergencies.

Emergency equipment availability remained particularly limited. Despite the workshop, the reported number of available equipment items showed little change, and participants continued to rate their equipment preparedness poorly. The low reported availability of oxygen cylinders and other emergency devices deserves particular attention in the context of Croatian regulatory requirements [31]. Although oxygen availability is required under Croatian regulations, only six participants reported having an oxygen cylinder in their dental practice. In primary healthcare institutions, dental practices may be located alongside family medicine practices, potentially allowing access to oxygen stored elsewhere in the building. However, the questionnaire did not assess shared access to emergency equipment or its immediate availability during an emergency. So, the low reported availability of oxygen cylinders in dental practices should not automatically be interpreted as a lack of access to oxygen, although reliance on shared resources may raise concerns regarding timely access in an emergency. The questionnaire assessed individually reported resources rather than verified regulatory compliance. Therefore, these findings cannot establish whether the participating practices met all applicable legal requirements. Nevertheless, they identify a potential patient-safety concern that warrants further investigation through direct assessment of equipment availability and functionality.

At baseline, dentists in the rural institution reported higher numbers of available medications and equipment items than those in the urban institution. Furthermore, the increase in self-perceived educational adequacy was greater in the rural group, whereas changes in medication and equipment availability did not differ significantly between settings. These findings should be interpreted cautiously. Each geographical setting was represented by only one institution, making it impossible to distinguish geographical characteristics from institutional differences. Although the educational content, instructors, and practical equipment were standardized across the two workshops, unmeasured institutional characteristics, organizational resources, and previous educational experiences may have influenced the results. The urban–rural comparison was therefore exploratory and cannot establish a general geographical pattern in emergency preparedness.

The findings should also be considered in relation to the previous Croatian national study by Špiljak et al. [29], which documented variability in dentists’ self-perceived readiness and the availability of emergency medications and equipment. While that study provided a broader cross-sectional overview of preparedness in Croatian dental practice, the present research adds paired observations collected before and one month after a practical educational intervention. However, the substantially smaller sample and the inclusion of only two institutions limit the generalizability of the present findings. Future research combining multi-centre recruitment, objective assessment of emergency resources, and standardized measures of clinical performance would provide a stronger basis for evaluating educational interventions and potential geographical differences.

The relatively high number of previously encountered cases of status epilepticus reported by participants in the rural institution warrants caution. Although status epilepticus was explicitly listed in the questionnaire, the reported diagnoses were not clinically verified and may reflect differences in interpretation or recall. Furthermore, these events referred to participants’ entire previous clinical experience rather than the one-month follow-up period. Consequently, the observed between-group differences should not be interpreted as evidence of a higher incidence of status epilepticus in rural dental practice.

Several limitations should be acknowledged. First, the absence of a control group prevents causal attribution of the observed changes to the workshop. Second, the small convenience sample, drawn from two institutions, limits statistical precision and generalizability. The geographical setting was completely confounded with institution, and each workshop was delivered at a different site. Third, the questionnaire was developed specifically for this study and underwent preliminary pilot testing but was not formally psychometrically validated. All outcomes were self-reported, introducing potential recall, response, and social desirability biases. Medication and equipment availability was not independently verified, and no information was collected on whether reported changes represented new purchases or increased recognition of existing resources. Additionally, the one-month follow-up did not allow assessment of long-term knowledge or skill retention. Finally, no formal a priori sample size calculation was performed, and the study may have had limited precision for detecting smaller changes or differences between settings.

Despite these limitations, the study provides paired observations on both dentists’ perceptions and practice-level resources following a standardized educational workshop. The findings support the importance of evaluating these components separately rather than relying on a single measure of emergency preparedness. Future controlled, multi-centre studies should incorporate objective assessments of knowledge and practical skills, verified inventories of emergency medications and equipment, and longer follow-up periods to assess the sustainability and practical relevance of observed changes.

## 5. Conclusions

One month after a practical medical emergency workshop, dentists from two Croatian primary healthcare institutions reported improved self-perceived educational adequacy and increased reported availability of emergency medications, while perceived medication preparedness and both perceived and reported equipment preparedness remained largely unchanged. These findings highlight the importance of distinguishing between self-perceived preparedness and reported availability of emergency resources. However, the uncontrolled study design does not allow the observed changes to be attributed directly to the educational intervention.

The greater increase in self-perceived educational adequacy observed in the rural institution should be considered exploratory, as geographical setting could not be separated from institution-specific characteristics. The findings suggest that practical training and appropriate emergency resources should be considered complementary components of preparedness. Larger, controlled, multi-centre studies incorporating objective assessments are needed to establish the effectiveness of educational interventions and inform recommendations for emergency preparedness in primary dental care.

## Figures and Tables

**Table 1:** Baseline characteristics and previous clinical experience with medical emergencies.

Characteristic	Urban (*n* = 18)	Rural (*n* = 24)	Overall (*N* = 42)	*p* Value
Age, years, median [IQR]	38.5 [32.8–52.0]	38.0 [33.5–50.5]	38.5 [32.5–52.0]	0.929
Professional experience, years, median [IQR]	11 [7,8,9,10,11,12,13,14,15,16,17,18,19,20,21,22]	12 [7,8,9,10,11,12,13,14,15,16,17,18,19,20,21]	12 [7,8,9,10,11,12,13,14,15,16,17,18,19,20,21,22]	0.859
Female, *n* (%)	14 (77.8)	18 (75.0)	32 (76.2)	—
Medical emergency education during dental school, *n* (%)	7 (38.9)	9 (37.5)	16 (38.1)	1.000
Previous medical emergency training course, *n* (%)	4 (22.2)	5 (20.8)	9 (21.4)	1.000
Previously encountered ≥1 medical emergency, *n* (%)	9 (50.0)	13 (54.2)	22 (52.4)	1.000

Values are presented as median [IQR] or *n* (%). Between-group comparisons were performed using the Mann–Whitney U test for numerical variables and Fisher’s exact test for categorical variables. Previous education and experience were assessed at baseline. One response regarding previous training was ambiguous; percentages are presented using the full group denominators.

**Table 2:** Changes in self-perceived preparedness and reported emergency resources (*N* = 42).

Preparedness Dimension/Outcome	Before, Median [IQR]	One Month, Median [IQR]	Improved, *n* (%)	Wilcoxon *p*
Educational Preparedness				
Perceived adequacy of education (1–5)	2 [2,3]	4 [3,4]	33 (78.6)	<0.001
Medication Preparedness				
Perceived adequacy of medication supply (1–5)	2 [2,3]	3 [2,3]	9 (21.4)	0.415
Number of medications reported available (0–9)	3 [2,3,4]	4 [3–4.75]	15 (35.7)	<0.001
Equipment Preparedness				
Perceived adequacy of equipment (1–5)	1 [1,2]	1 [1,2]	5 (11.9)	0.499
Number of equipment items reported available (0–8)	0 [0–0]	0 [0–1]	3 (7.1)	0.083

Higher self-assessment scores indicate greater perceived adequacy. Improved denotes a positive individual follow-up minus baseline change. Medication and equipment counts are self-reported (not independently verified). The difference between reported supply and perceived sufficiency should not be interpreted causally.

**Table 3:** Exploratory urban–rural comparison of individual changes.

Outcome	Rural—Urban Mean Change	95% CI for Difference	*P* (Rural > Urban) *	95% CI for *P* *	Mann–Whitney *p*
Self-perceived educational adequacy (1–5)	+1.11	+0.68 to +1.53	0.843	0.733 to 0.931	<0.001
Perceived equipment adequacy (1–5)	−0.03	−0.40 to +0.39	0.454	0.316 to 0.595	0.544
Perceived medication adequacy (1–5)	+0.01	−0.56 to +0.57	0.512	0.353 to 0.667	0.897
Reported equipment items (0–8)	−0.07	−0.24 to +0.08	0.465	0.382 to 0.542	0.409
Reported medications (0–9)	+0.06	−0.36 to +0.50	0.488	0.339 to 0.635	0.893

* Common-language effect size: probability that a randomly selected rural participant has a larger individual change than an urban participant, counting ties as 0.5. Percentile bootstrap 95% confidence intervals were calculated using 20,000 resamples. As only one institution represented each setting, all between-group comparisons are descriptive and exploratory.

## Data Availability

The datasets generated and/or analysed during the current study are not publicly available because they contain participant-level data from a small professional sample, but are available from the corresponding author upon reasonable request.

## Supplementary Materials

The following supporting information can be downloaded at: https://www.mdpi.com/article/10.3390/healthcare14213695/s1, Table S1: Medical emergencies previously encountered by participating dentists; Table S2: Item-level changes in reported availability of emergency medications and equipment; File S1: Questionnaire.

## References

[1] Cangül S., Taşyürek M., Adıgüzel Ö., Aşır F. (2026). Knowledge, Attitudes, and Clinical Preparedness of Dentists for Medical Emergencies: A Nationwide Cross-Sectional Survey. Healthcare.

[2] Pius L., Brady N., Overby M., Zhu J., Ferraro N. (2023). Emergency protocol in the dental clinic: Assessing medical emergency training requirements and guidelines for dentists. J. Am. Dent. Assoc..

[3] Varoni E.M., Rigoni M., Lodi G., Sardella A., Muti P., Vitello A., Montebugnoli L., Polimeni A., Tommasino S., Iriti M. (2023). Medical emergencies in dental practice: A nationwide web-based survey of Italian dentists. Heliyon.

[4] Brailo V., Brailo V. (2024). Vazovagalna reakcija. Hitna medicinska stanja u dentalnoj medicini.

[5] Sooch S., Kaur A., Ahmed B. (2018). A ten year experience of medical emergencies at Birmingham Dental Hospital. Br. Dent. J..

[6] Zachar J.J., Reher P. (2022). Frequency and characteristics of medical emergencies in an Australian dental school: A retrospective study. J. Dent. Educ..

[7] Shao X., Bai J., Ji A., Sun W. (2023). Retrospective analysis of medical emergencies in an oral emergency department. Med. Oral Patol. Oral Cir. Bucal.

[8] Kishimoto N., Sanuki T., Liu Y., Tran S.D., Seo K. (2023). Simulation training for medical emergencies of dental patients: A review of the dental literature. Jpn. Dent. Sci. Rev..

[9] Smyth M.A., van Goor S., Hansen C.M., Fijačko N., Nakagawa N.K., Raffay V., Ristagno G., Rogers J., Scquizzato T., Smith C.M. (2025). European Resuscitation Council Guidelines 2025 Adult Basic Life Support. Resuscitation.

[10] Resuscitation Council UK Quality Standards: Primary Dental Care Equipment List; Updated May 2020. https://www.resus.org.uk/library/quality-standards-cpr/primary-dental-care-equipment-list.

[11] Smereka J., Aluchna M., Aluchna A., Szarpak Ł. (2019). Preparedness and attitudes towards medical emergencies in the dental office among Polish dentists. Int. Dent. J..

[12] Marks L.A., Van Parys C., Coppens M., Herregods L. (2013). Awareness of dental practitioners to cope with a medical emergency: A survey in Belgium. Int. Dent. J..

[13] Abdulrahman B.I., Alasmari K.M., Alratiq M.N., Alherab F.A., Alfantoukh M.A., Alherab A.A. (2022). Are dental professionals ready to deal with medical emergencies in their clinical office? A survey of university hospitals. J. Med. Life..

[14] Gupta S., Mishra S., Behl S., Srikant N., Mascarenhas R. (2023). Knowledge of handling medical emergencies among general dental practitioners pan India: A cross-sectional survey. BMC Res. Notes.

[15] Murariu A., Bobu L., Stoleriu S., Vasluianu R.I., Iovan G., Gelețu G.L., Toma V., Baciu E.R. (2024). Knowledge and Attitudes towards Medical Emergencies among Dentists in Iași, Romania. Dent. J..

[16] Shaath H., Salman B., Daghistani D., Koutaich R., Alhammadi A., Yakoub N., Awad M.A. (2023). A Pilot Study of Preparedness of Dentists in the United Arab Emirates to Deal with Medical Emergencies. Eur. J. Dent..

[17] Cooper S., Johnston E., Priscott D. (2007). Immediate life support (ILS) training Impact in a primary care setting?. Resuscitation.

[18] Kovács E., Jenei Z.M., Csordás K., Fritúz G., Hauser B., Gyarmathy V.A., Zima E., Gál J. (2019). The timing of testing influences skill retention after basic life support training: A prospective quasi-experimental study. BMC Med. Educ..

[19] Cho Y., Je S., Yoon Y.S., Roh H.R., Chang C., Kang H., Lim T. (2016). The effect of peer-group size on the delivery of feedback in basic life support refresher training: A cluster randomized controlled trial. BMC Med. Educ..

[20] Kochhar S., Samagh N., Sharma J., Kaur A., Verma M. (2024). Knowledge and Skill Retention in First-Year MBBS Students After Basic Life Support Training: A One-Year Longitudinal Study. BMC Med. Educ..

[21] Müller M.P., Hänsel M., Stehr S.N., Weber S., Koch T. (2008). A state-wide survey of medical emergency management in dental practices: Incidence of emergencies and training experience. Emerg. Med. J..

[22] Solanki C., Geisinger M.L., Luepke P.G., Al-Bitar K., Palomo L., Lee W., Blanchard S., Shin D., Maupome G., Eckert G.J. (2021). Assessing readiness to manage medical emergencies among dental students at four dental schools. J. Dent. Educ..

[23] Kishimoto N., Ujita T., Tran S.D., Sanuki T., Seo K. (2024). Simulation training for medical emergencies: Evaluation of dentists’ long-term learning skills and confidence. Eur. J. Dent. Educ..

[24] Iacobescu R.-A., Blaga T., Dragomir R., Mihai Ș.-C., Moroșan P., Hăisan A. (2026). Medical Emergencies and Operational Preparedness Among Dentists: A Scoping Review. Dent. J..

[25] Alawaji Y.N., Alharthy N.A., Alghamdi A., Alshibani A., Binhotan M., Alqahtani H., Albalawi F., Alshehri A., Aboalela A., Altuwaijri S.H. (2026). Effectiveness of Scenario-Based High-Fidelity Simulation Training on Management of Medical Emergencies in Dental Settings: A Theory-Based Intervention with a Long-Term Follow-Up. BMC Med. Educ..

[26] Luo H., Wu Q., Bell R.A., Wright W., Quandt S.A., Basu R., Moss M.E. (2021). Rural-Urban Differences in Dental Service Utilization and Dental Service Procedures Received Among US Adults: Results From the 2016 Medical Expenditure Panel Survey. J. Rural Health.

[27] Jo O., Kruger E., Tennant M. (2020). Geospatial analysis of the urban and rural/remote distribution of dental services in Scotland, Wales and Northern Ireland. Int. Dent. J..

[28] Kaddah M., Alkhouri I., Alkhalaf E. (2025). Medical Emergency Preparedness of Dental Clinics in War-Torn Syria: A Cross-Sectional Study. Sci. Rep..

[29] Špiljak B., Firić M., Sikora M., Brailo V. (2024). Self-Assessment of Readiness and Equipment of Dental Clinics for Managing Medical Emergencies in the Republic of Croatia. Acta Stomatol. Croat..

[30] Alruwaili A., Alanazy A.R.M. (2022). Prehospital Time Interval for Urban and Rural Emergency Medical Services: A Systematic Literature Review. Healthcare.

[31] Ministry of Health of the Republic of Croatia Regulation on Norms and Standards for the Provision of Healthcare Services. https://narodne-novine.nn.hr/clanci/sluzbeni/2020_04_52_1048.html.

