# Supplementary material for: Medical Emergency Preparedness in Primary Dental Care Following Practical Training: An Urban–Rural Comparison
Source: Healthcare (Basel). 2026 Nov 1;14(21):3695. doi: 10.3390/healthcare14213695 (PMC13763281; doi:10.3390/healthcare14213695)
Supplement: Supplementary file 1 [file healthcare-14-03695-s001.zip › Supplementary File S1 Questionnaire-edited.pdf]

Supplementary File S1

Questionnaire

Dentists' Competencies and Emergency Equipment in Dental Practices

English translation of the original Croatian questionnaire (5 June 2023). Question numbering, response options, and skip instructions have been preserved. Select one answer unless otherwise indicated.

1. Sex (circle one):

a) Male
b) Female

2. Enter your age (years): _________

3. How many years have you worked in a dental practice? _________

4. Specialty:

a) I do not have a specialty
b) Specialist in oral medicine
c) Specialist in oral surgery
d) Specialist in endodontics and restorative dentistry
e) Specialist in paediatric dentistry
f) Specialist in family dentistry
g) Specialist in orthodontics
h) Specialist in periodontology
i) Specialist in prosthodontics

5. In which Croatian county is your dental practice located? (circle one)

1. Bjelovar-Bilogora County
2. Brod-Posavina County
3. Dubrovnik-Neretva County
4. Istria County
5. Karlovac County
6. Koprivnica-Križevci County
7. Krapina-Zagorje County
8. Lika-Senj County
9. Međimurje County
10. Osijek-Baranja County
11. Požega-Slavonia County
12. Primorje-Gorski Kotar County
13. Sisak-Moslavina County
14. Split-Dalmatia County
15. Varaždin County
16. Virovitica-Podravina County

17. Vukovar-Srijem County

18. Zadar County

19. Zagreb County

20. Šibenik-Knin County

21. City of Zagreb

6. What is the population of the town or settlement where your dental practice is located?

a) Fewer than 5,000 inhabitants
b) 5,000–10,000 inhabitants
c) 10,000–50,000 inhabitants
d) 50,000–100,000 inhabitants
e) 100,000–500,000 inhabitants
f) More than 500,000 inhabitants

7. What is your professional employment/practice status? (circle one)

a) I work as a Croatian Health Insurance Fund (CHIF)-contracted dentist in a practice belonging to a health centre
b) I work as a CHIF-contracted dentist in a concession practice located within a health centre
c) I work as a CHIF-contracted dentist in a concession practice located in private premises
d) I work as a private dentist in private premises
e) I work as a dentist in a dental polyclinic
f) I work as a dentist in a clinical hospital
g) I work as a dentist in a university hospital centre
h) I work as a dentist at the School of Dental Medicine

8. During your studies at the School of Dental Medicine, did you take a course entitled "Medical Emergencies in Dental Practice" or a similar course, such as "Emergency Medicine" or "Emergencies in Dental Medicine"? (circle one)

a) Yes
b) No

9. During your studies or professional career, have you attended and completed any emergency medicine training course? (circle one)

a) Yes
b) No

10. If you answered "Yes" to Question 9, which emergency medicine course did you attend and complete? (write the course name): ________________________

11. Have you encountered any type of medical emergency during your work in a dental practice? (circle one)

a) Yes
b) No

12. If you answered "Yes" to Question 11, which types of medical emergencies have you encountered in your dental practice? (multiple answers allowed)

a) Chest pain (stable angina pectoris and acute coronary syndrome—myocardial infarction)

b) Cardiac arrest
c) Altered consciousness (syncope)
d) Acute heart failure and pulmonary oedema
e) Pulmonary embolism
f) Hypertensive reaction/crisis
g) Hypoglycaemia/diabetic ketoacidosis (coma)
h) Chronic obstructive pulmonary disease (COPD)/bronchial asthma
i) Anxiety
j) Reaction to local anaesthetic
k) Status epilepticus
l) Anaphylactic shock
m) Foreign-body aspiration

13. If you answered "Yes" to Question 11, how did you manage the medical emergency in your dental practice? (circle one)

a) I managed the medical emergency independently
b) I called a colleague from family medicine for assistance
c) I immediately called emergency medical services (194) to the practice
d) I referred the patient from the practice to the nearest emergency department

14. If you answered "Yes" to Question 11, how did you feel when the medical emergency occurred in the practice? (circle one)

a) Fear, panic, freezing
b) Calmness, composure, rational thinking

15. Which of the following equipment items are available in your dental practice? (multiple answers allowed)

a) Portable ECG device with a defibrillator or automated external defibrillator (AED)
b) Pulse oximeter
c) Magill forceps
d) Self-inflating bag with mask
e) Oropharyngeal airway
f) Endotracheal tube
g) Laryngoscope
h) Oxygen cylinder

16. Which of the following medications are available in your dental practice? (multiple answers allowed)

a) Adrenaline
b) Bronchodilators
c) Antihistamines
d) Nitroglycerin
e) Benzodiazepines
f) Acetylsalicylic acid
g) Glucose
h) Corticosteroids
i) Oxygen

17. How would you rate your own education (knowledge) in emergency medicine for providing emergency care in your dental practice?

a) Excellent
b) Very good
c) Good
d) Sufficient
e) Insufficient

18. How would you rate the adequacy of emergency equipment available in your dental practice?

a) Excellent
b) Very good
c) Good
d) Sufficient
e) Insufficient

19. How would you rate the adequacy of medications available for medical emergencies in your dental practice?

a) Excellent
b) Very good
c) Good
d) Sufficient
e) Insufficient

Translator's note: The original response options for Questions 17–19 were listed in descending order (Excellent to Insufficient). The numerical coding used in the manuscript is 1 = Insufficient to 5 = Excellent. "Oxygen cylinder" (Question 15) and "oxygen" (Question 16) are separate options in the original questionnaire.
