# Supplementary material for: Medical Emergency Preparedness in Primary Dental Care Following Practical Training: An Urban–Rural Comparison
Source: Healthcare (Basel). 2026 Nov 1;14(21):3695. doi: 10.3390/healthcare14213695 (PMC13763281; doi:10.3390/healthcare14213695)
Supplement: Supplementary file 1 [file healthcare-14-03695-s001.zip › Supplementary tables-proof.pdf]

Supplementary Table S1. Medical emergencies previously encountered by participating dentists.

Previously encountered emergency	Urban (n = 18)	Rural (n = 24)	Overall (N = 42)
Syncope	7 (38.9)	8 (33.3)	15 (35.7)
Anxiety-related event	4 (22.2)	9 (37.5)	13 (31.0)
Reaction associated with local anaesthetic	5 (27.8)	5 (20.8)	10 (23.8)
Status epilepticus (questionnaire wording)	0 (0.0)	6 (25.0)	6 (14.3)
Hypoglycaemia/diabetic ketoacidosis	0 (0.0)	4 (16.7)	4 (9.5)
Hypertensive crisis	0 (0.0)	3 (12.5)	3 (7.1)
Chest pain (angina/acute coronary syndrome)	0 (0.0)	2 (8.3)	2 (4.8)

Values are n (%); multiple answers were permitted. Retrospective self-report without a fixed observation period or clinical verification. "Status epilepticus" is the exact original questionnaire label and should not be interpreted as clinically adjudicated status epilepticus.

Supplementary Table S2. Item-level changes in reported availability of emergency medications and equipment.

Item	Baseline n (%)	One month n (%)	Unavailable → available	Available → unavailable	Exact McNemar p	Holm-adjusted p
▸ **EMERGENCY MEDICATIONS**						
Adrenaline	41 (97.6)	41 (97.6)	0	0	1.000	1.000
Bronchodilators	13 (31.0)	18 (42.9)	5	0	0.0625	0.500
Antihistamines	39 (92.9)	39 (92.9)	0	0	1.000	1.000
Nitroglycerin	2 (4.8)	3 (7.1)	1	0	1.000	1.000
Benzodiazepines	7 (16.7)	7 (16.7)	1	1	1.000	1.000
Acetylsalicylic acid	4 (9.5)	6 (14.3)	2	0	0.500	1.000
Glucose	5 (11.9)	8 (19.0)	4	1	0.375	1.000
Corticosteroids	28 (66.7)	37 (88.1)	9	0	0.0039	0.035
Oxygen	5 (11.9)	5 (11.9)	0	0	1.000	1.000
▸ **EMERGENCY EQUIPMENT**						
Automated external defibrillator	0 (0.0)	2 (4.8)	2	0	0.500	1.000
Pulse oximeter	8 (19.0)	8 (19.0)	0	0	1.000	1.000
Magill forceps	0 (0.0)	0 (0.0)	0	0	1.000	1.000
Self-inflating bag and mask	5 (11.9)	5 (11.9)	0	0	1.000	1.000
Oropharyngeal airway	0 (0.0)	1 (2.4)	1	0	1.000	1.000
Endotracheal tube	1 (2.4)	1 (2.4)	0	0	1.000	1.000
Laryngoscope	0 (0.0)	0 (0.0)	0	0	1.000	1.000
Oxygen cylinder	6 (14.3)	6 (14.3)	0	0	1.000	1.000

All proportions use N = 42 paired dentists. Discordant pairs denote changes in reported availability, not documented purchases/disposals. Exact two-sided McNemar tests; Holm step-down adjustment performed separately within nine medications and eight equipment items. Oxygen listed under medications and oxygen cylinder under equipment reflect separate questions, not independent confirmation of oxygen provision.
